# Arthroscopic hip surgery compared with physiotherapy and activity modification for the treatment of symptomatic femoroacetabular impingement: multicentre randomised controlled trial

**DOI:** 10.1136/bmj.l185

**Published:** 2019-02-07

**Authors:** Antony J R Palmer, Vandana Ayyar Gupta, Scott Fernquest, Ines Rombach, Susan J Dutton, Ramy Mansour, Simon Wood, Vikas Khanduja, Tom C B Pollard, Andrew W McCaskie, Karen L Barker, Tony J M D Andrade, Andrew J Carr, David J Beard, Sion Glyn-Jones

**Affiliations:** 1 Nuffield Department of Orthopaedics, Rheumatology, and Musculoskeletal Sciences (NDORMS), University of Oxford, Botnar Research Centre, Oxford OX3 7LD, UK; 2 Centre for Statistics in Medicine, NDORMS, University of Oxford, UK; 3 Oxford University Hospitals NHS Foundation Trust, Oxford, UK; 4 Cambridge University Hospitals NHS Foundation Trust, Cambridge, UK; 5 Royal Berkshire NHS Foundation Trust, Reading, UK; 6 Division of Trauma and Orthopaedic Surgery, University of Cambridge, UK; 7 Royal College of Surgeons Surgical Intervention Trials Unit, NDORMS, University of Oxford, Oxford, UK

## Abstract

**Objective:**

To compare arthroscopic hip surgery with physiotherapy and activity modification for improving patient reported outcome measures in patients with symptomatic femoroacetabular impingement (FAI).

**Design:**

Two group parallel, assessor blinded, pragmatic randomised controlled trial.

**Setting:**

Secondary and tertiary care centres across seven NHS England sites.

**Participants:**

222 participants aged 18 to 60 years with symptomatic FAI confirmed clinically and with imaging (radiography or magnetic resonance imaging) were randomised (1:1) to receive arthroscopic hip surgery (n=112) or a programme of physiotherapy and activity modification (n=110). Exclusion criteria included previous surgery, completion of a physiotherapy programme targeting FAI within the preceding 12 months, established osteoarthritis (Kellgren-Lawrence grade ≥2), and hip dysplasia (centre-edge angle <20 degrees).

**Interventions:**

Participants in the physiotherapy group received a goal based programme tailored to individual patient needs, with emphasis on improving core stability and movement control. A maximum of eight physiotherapy sessions were delivered over five months. Participants in the arthroscopic surgery group received surgery to excise the bone that impinged during hip movements, followed by routine postoperative care.

**Main outcome measures:**

The primary outcome measure was the hip outcome score activities of daily living subscale (HOS ADL) at eight months post-randomisation, with a minimum clinically important difference between groups of 9 points. Secondary outcome measures included additional patient reported outcome measures and clinical assessment.

**Results:**

At eight months post-randomisation, data were available for 100 patients in the arthroscopic hip surgery group (89%) and 88 patients in the physiotherapy programme group (80%). Mean HOS ADL was 78.4 (95% confidence interval 74.4 to 82.3) for patients randomised to arthroscopic hip surgery and 69.2 (65.2 to 73.3) for patients randomised to the physiotherapy programme. After adjusting for baseline HOS ADL, age, sex, and study site, the mean HOS ADL was 10.0 points higher (6.4 to 13.6) in the arthroscopic hip surgery group compared with the physiotherapy programme group (P<0.001)). No serious adverse events were reported in either group.

**Conclusions:**

Patients with symptomatic FAI referred to secondary or tertiary care achieve superior outcomes with arthroscopic hip surgery than with physiotherapy and activity modification.

**Trial registration:**

ClinicalTrials.gov NCT01893034.

## Introduction

**Fig 1:**
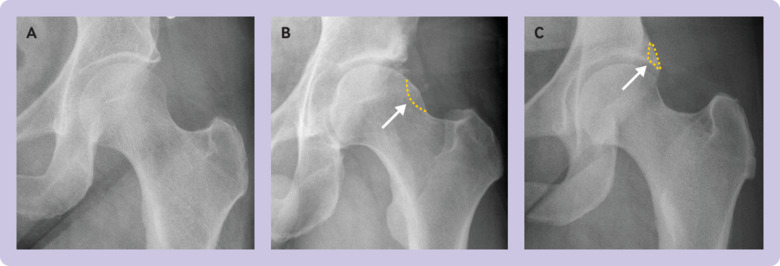
Anteroposterior radiographs showing (A) normal morphology, (B) cam morphology, and (C) pincer morphology. Dashes represent abnormal morphology that predisposes to femoroacetabular impingement, and which is excised with a burr during arthroscopic surgery to prevent impingement

Femoroacetabular impingement (FAI) is a hip condition where adverse morphology predisposes to premature joint degeneration.^1^ ^2^ This adverse morphology is classified as cam, pincer, or mixed. Cam morphology describes a loss of sphericity of the femoral head, pincer morphology describes an acetabulum with excessive coverage of the femoral head, and mixed morphology describes a combination of the two deformities (fig 1). These hip shapes can cause the femoral neck to impact against the acetabular rim during a functional range of movement, with resultant damage to the labrum (which is attached to the rim), delamination of the adjacent acetabular cartilage, and, over time, secondary osteoarthritis.^1^ ^3^

The prevalence of FAI morphology is high and is observed in about one fifth of the general population.^4^ Less than 25% of those affected develop pain^5^ (FAI syndrome) or osteoarthritis,^1^ although up to 50% of all hip osteoarthritis might develop secondary to FAI.^2^ Identifying those at greatest risk of developing joint disease secondary to FAI remains a challenge.

**Fig 2:**
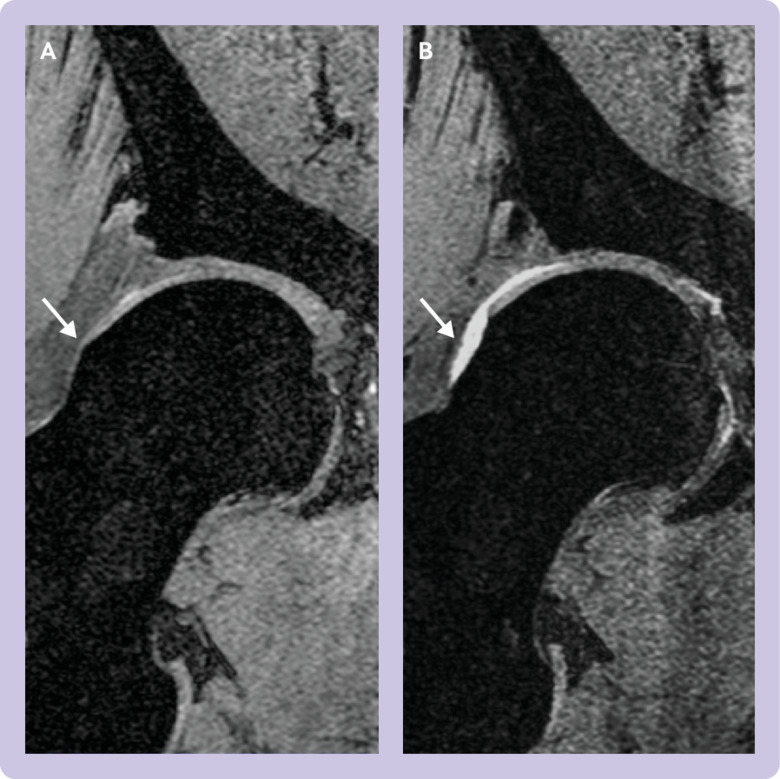
Right hip coronal magnetic resonance image of trial participant randomised to surgery: (A) Baseline image showing cam morphology (arrow). (B) Six months after hip arthroscopy with restoration of the normal concavity at the femoral head-neck junction by burring away the cam lesion (arrow). This procedure prevents abutment of the femoral head-neck junction against the acetabular rim during a functional range of movement

Physiotherapy and activity modification represents the principal treatment for symptomatic FAI; however, arthroscopic surgery is increasingly adopted to reshape the hip and deal with the damage to the labrum and cartilage (fig 2). The primary treatment goal is to improve pain and function, but interventions that modify contact between the femoral neck and acetabular rim may subsequently reduce cartilage and joint damage, the risk of osteoarthritis, and need for future hip arthroplasty.^6^

Although arthroscopic hip surgery has been shown to be safe,^7^ evidence of efficacy is limited. Two randomised controlled trials compared physiotherapy rehabilitation with arthroscopy for improving symptoms: one concluded that treatments did not differ^8^ and the other that arthroscopic surgery was superior to best conservative care.^9^ Despite the limited evidence, arthroscopic hip surgery has become an established treatment, with an estimated 50 000 or more procedures being carried out in the United States annually.^10^ The number of procedures performed annually in England between 2002 and 2013 increased by 727%.^11^ Regional variation in the number of procedures performed is substantial and could reflect surgeon preference or local commissioning of services.^11^

The Femoroacetabular Impingement Trial (FAIT) compared arthroscopic hip surgery with physiotherapy and activity modification in patients referred to secondary or tertiary care with symptomatic FAI.^12^ Here we report the primary endpoint of patient reported outcomes at eight months post-randomisation. Cost effectiveness and development of osteoarthritis will be evaluated at three year follow-up. The study design was based on a previous feasibility study, which showed that both surgeons and patients have equipoise for physiotherapy and activity modification versus arthroscopic hip surgery.^13^

## Methods

The study was performed according to the published protocol.^12^ FAIT is a two group parallel assessor blinded pragmatic randomised controlled study with 1:1 allocation.

### Participants

Eligible participants were aged 18 to 60 years and referred to secondary or tertiary care with symptomatic FAI confirmed clinically and with imaging (radiography and magnetic resonance imaging (MRI)). Owing to the absence of agreed diagnostic thresholds and to improve generalisability of our study findings, we did not use quantitative imaging measurements as inclusion criteria for this study.^14^ Instead, surgeons qualitatively assessed hip morphology to diagnose FAI. We excluded participants if they had completed a programme of physiotherapy targeting FAI within the preceding 12 months or received previous surgery to their symptomatic hip. Additional exclusion criteria were established osteoarthritis (Kellgren-Lawrence grade ≥2) or hip dysplasia (centre-edge angle <20 degrees on anteroposterior pelvis radiograph).

### Recruiting centres

Consultant orthopaedic surgeons from seven National Health Service sites across England recruited participants: Oxford University Hospitals NHS Foundation Trust, Royal Berkshire NHS Foundation Trust, Harrogate and District NHS Foundation Trust, Cambridge University Hospital NHS Foundation Trust, Wye Valley NHS Trust, Great Western Hospital NHS Foundation Trust, and Weston Area Health NHS Trust. Study participation required that sites were centres that perform a high volume of arthroscopic hip procedures and could deliver the goal based physiotherapy programme.

### Randomisation and masking

A research nurse at each site performed randomisation using an automated computer generated telephone randomisation system provided by the Oxford Clinical Trials Research Unit. Randomisation for the first 12 participants (10% of original sample size) was based on a simple random list, and a minimisation algorithm was used to randomise subsequent participants. This algorithm included a random element (80%) and aimed to generate balanced treatment allocations by age (<40 or ≥40 years), sex, baseline activities of daily living subscale of the hip outcome score (HOS ADL) (<65% or ≥65%), and study site.^12^

It was not possible to mask participants, or clinicians delivering the intervention. However, clinicians performing follow-up clinical assessments (hip range of movement and impingement tests) were blinded to the treatment group. Participants were asked to not disclose their treatment and to wear shorts to cover any scars. Staff members independent of the study team carried out data entry.

### Interventions

Full details of the interventions are in the published protocol.^13^

*Physiotherapy and activity modification*—as no standardised physiotherapy regimen has been agreed for FAI, we developed a goal based programme based on the consensus opinion of the study team and existing literature.^15^ To standardise treatment, participating physiotherapists received information on the study protocol and training sessions. The treating therapist recorded physiotherapy compliance and attainment of goals within the prescribed treatment themes. A specialist physiotherapist (band 6) or advanced physiotherapy practitioner (band 7/8) delivered the treatment (supplementary table S1). The programme was tailored to individual patient needs and desired level of function, with an emphasis on muscle strengthening to improve core stability and movement control. Participants were encouraged to avoid impingement positions (extremes of hip flexion, abduction, internal rotation). To reflect what is feasible in current NHS practice, we provided a maximum of eight sessions over a five month period.

*Arthroscopic surgery*—before trial recruitment began, participating surgeons met to ensure standardisation of technique for the study by consensus agreement. Femoral and acetabular bone seen to impinge intraoperatively were excised with a burr (osteochondroplasty) to eliminate impingement on dynamic hip flexion and internal rotation. Labral tears were repaired if possible, or otherwise debrided. Articular cartilage lesions were debrided to a stable base, and in areas of full thickness cartilage loss, microfracture of the subchondral bone was performed. Participants received postoperative physiotherapy, provided as routine care in the NHS, which focused on maintaining range of movement and a graduated return to activity.

### Outcomes

The primary outcome measure was the HOS ADL (range 0 to 100, with higher values indicating better outcomes) at eight months post-randomisation. The HOS ADL is a validated patient reported outcome measure for arthroscopic hip procedures.^16^

Secondary outcomes were additional patient reported outcome measures on symptoms: HOS sport subscale,^16^ non-arthritic hip score (NAHS),^17^ Copenhagen hip and groin outcome score (HAGOS),^18^ Oxford hip score (OHS),^19^ and international hip outcome tool (iHOT-33).^20^ Quality of life, nature and location of pain, and psychological factors were evaluated using EQ-5D-3L,^21^ PainDETECT,^22^ and hospital anxiety and depression score (HADS),^23^ respectively. At baseline, participants were also asked to complete an “expectation” HOS ADL to indicate the symptoms they expected to experience after completion of treatment.

Clinical assessment performed at baseline and follow-up visits consisted of range of passive hip movement, measured using a goniometer, and recording whether a participant experienced pain on each movement. Impingement tests determined whether a participant experienced pain on hip flexion, adduction, and internal rotation (FADIR) or flexion, abduction, and external rotation (FABER).

Academic orthopaedic clinicians (AJRP and SF) used custom software to carry out imaging measurements. Osteoarthritis was evaluated using the Kellgren-Lawrence grading classification.^24^ Dysplasia and pincer morphology were quantified using the centre-edge angle on a standing anteroposterior radiograph. Cam morphology was measured as the maximal cartilage α angle at the 12 o’clock, 1 o’clock, 2 o’clock, and 3 o’clock position on MRI radial slices.^25^ All intraclass correlation coefficients for intra-observer and interobserver reproducibility values exceeded 0.90, suggesting excellent agreement (supplementary fig S1). 

Participants will be followed up for three years to evaluate the development of osteoarthritis in this cohort. Additional outcomes (not reported here) for the long term analysis include compositional MRI (T2 mapping), serum and urinary biomarkers of osteoarthritis, and health economic data.^12^

### Study assessments

We collected the primary and secondary outcome measures at baseline and eight months after randomisation, equating to approximately six months after intervention when accounting for waiting times to treatment. This time point was chosen because a clinically meaningful difference of 9 points in the HOS ADL is detectable six months after arthroscopic hip surgery,^16^ ^26^ and our feasibility study found that 94% of patients were willing to pursue a treatment of six months, but no longer, without improvement in symptoms.^13^

If treatment commenced more than 12 weeks post-randomisation, follow-up assessments were performed six months post-intervention rather than eight months post-randomisation to ensure the schedule remained aligned with routine clinical care. We collected patient reported outcome measures at eight months post-randomisation (primary outcome measure) and six months post-intervention in this group.

### Sample size

Sample size was based on the primary outcome measure, HOS ADL at eight months post-randomisation, and was calculated using a minimum clinically important difference between groups of 9 points.^16^ We estimated the standard deviation to be 14 points; however, summaries presented at a planned interim data monitoring meeting found that the standard deviation was 18 points. A revised calculation (significance level 5%, power 90%, loss to follow-up 20%) gave a sample size of 214 (107 participants in each group). The data monitoring committee approved the sample size increase from 120 to 214 participants.

### Statistical analysis

The statistical analysis plan was finalised before unblinding of data to study investigators. Statistical testing was performed at the two sided 5% significance level and conducted using STATA 14.2 (StataCorp LLC, College Station, TX). Analysis of the primary endpoint and all secondary endpoints was according to modified intention to treat (mITT), including patients with available outcome data based on their randomised treatment allocation, regardless of compliance. We used linear regression analysis to compare the HOS ADL outcomes at eight months post-randomisation between the treatment groups, adjusting for the minimisation factors sex, age, baseline HOS ADL, and site (using cluster robust standard errors, implemented via the cluster option in Stata). Results are presented as treatment effects with 95% confidence intervals and P values.

In addition to HOS ADL evaluation within the cohort, we also assessed HOS ADL within individuals, expressed as the proportion of patients achieving: an increase in HOS ADL greater than 9 points (minimum detectable change and a clinically important change within an individual),^16^ a patient acceptable symptomatic state (PASS) (outcome HOS ADL ≥87 points)^27^ within the mITT population eight months post-randomisation, and an expectation HOS ADL (the score patients expect to achieve after treatment measured at baseline).

Supporting analyses of the primary endpoint included a multilevel mixed effects model with repeated measures of HOS ADL, adjusting for baseline HOS ADL, sex, age, time from randomisation, and study site (analysis A). The primary analysis was then repeated with additional adjustment for HADS, imaging measures of osteoarthritis (radiographic Kellgren-Lawrence grade), hip morphology (maximum cartilage α angle on MRI, and centre-edge angle on anteroposterior pelvis radiograph) (analysis B); the per protocol population, excluding participants with major deviations from the trial protocol (analysis C); and six months post-intervention outcomes (analysis D). We also repeated the primary analysis with the baseline expectation HOS ADL as a covariate. Participants with available baseline and outcome data were included in these analyses.

To consider the potential impact of missing data on trial conclusions, we used multiple imputation (data missing at random) and sensitivity analysis (data not missing at random). Multiple imputation by chained equations was performed using the “mi impute chained” command in Stata. We used a linear regression model to impute missing outcomes for the HOS ADL at eight months post-randomisation. Variables in the imputation model included all covariates in the analysis model (baseline HOS ADL (continuous), age (continuous), and sex). In addition, we included other variables that were thought to be predictive of the outcome (lateral centre-edge angle, maximum α angle, Kellgren-Lawrence grade, and baseline HADS score). Imputations were run separately by treatment arm and based on a predictive mean matching approach, choosing at random one of the five HOS ADL values with the closest predicted scores. Missing data in the covariates that were included in the multiple imputation model were imputed simultaneously (multiple imputation by chained equation approach). Sensitivity analysis was performed using the “rctmiss” command in Stata, and we considered scenarios where participants with missing data in each arm were assumed to have outcomes that were up to 9 points worse than when data were missing at random (supplementary fig S2).

We used a multilevel mixed effects model to analyse secondary patient reported outcome measures, with repeated measures of the relevant patient reported outcome measures (collected at five and eight months) nested within participants. The models used data from participants with available baseline information and at least one follow-up assessment, adjusted for baseline patient reported outcome measure, sex, age, study site, and time from randomisation.

Predefined subgroup exploration was performed for several participant groups: osteoarthritis severity (Kellgren-Lawrence grade 0 *v* 1), sex, age (continuous variable), baseline HOS ADL (continuous variable), and FAI type (pincer, cam, or mixed). Treatment effects by binary subgroup were illustrated with forest plots, showing point estimates, confidence intervals, and heterogeneity P values (estimates obtained from interaction models including only the relevant subgroup and randomised treatment as covariates). We explored the differential treatment effect for age and baseline HOS ADL (as continuous variables) by adding an interaction term for treatment×age and treatment×baseline HOS ADL into the primary analysis model. Linear and non-linear effects (squared and cubic terms) for age and baseline HOS ADL were explored.

For each follow-up time point we summarised descriptively the details on clinical examination, including range of movement and signs of impingement. Differences in range of movement between the treatment groups were obtained from linear regression models adjusted for baseline values. Differences between treatment groups were explored using χ^2^ tests for signs of impingement.

### Patient and public involvement

A feasibility study included patient questionnaires to determine outcomes they thought were most important, treatment preferences, acceptable study design, and anticipated recruitment numbers.^13^ The study design was based on these findings. A patient representative provided guidance throughout the study, including an evaluation of the burden of intervention and assessments. Study results will be disseminated through publication, presentation at scientific meetings, and at patient and public engagement events coordinated by our institution. The results will also be disseminated using social media platforms.

## Results

**Fig 3:**
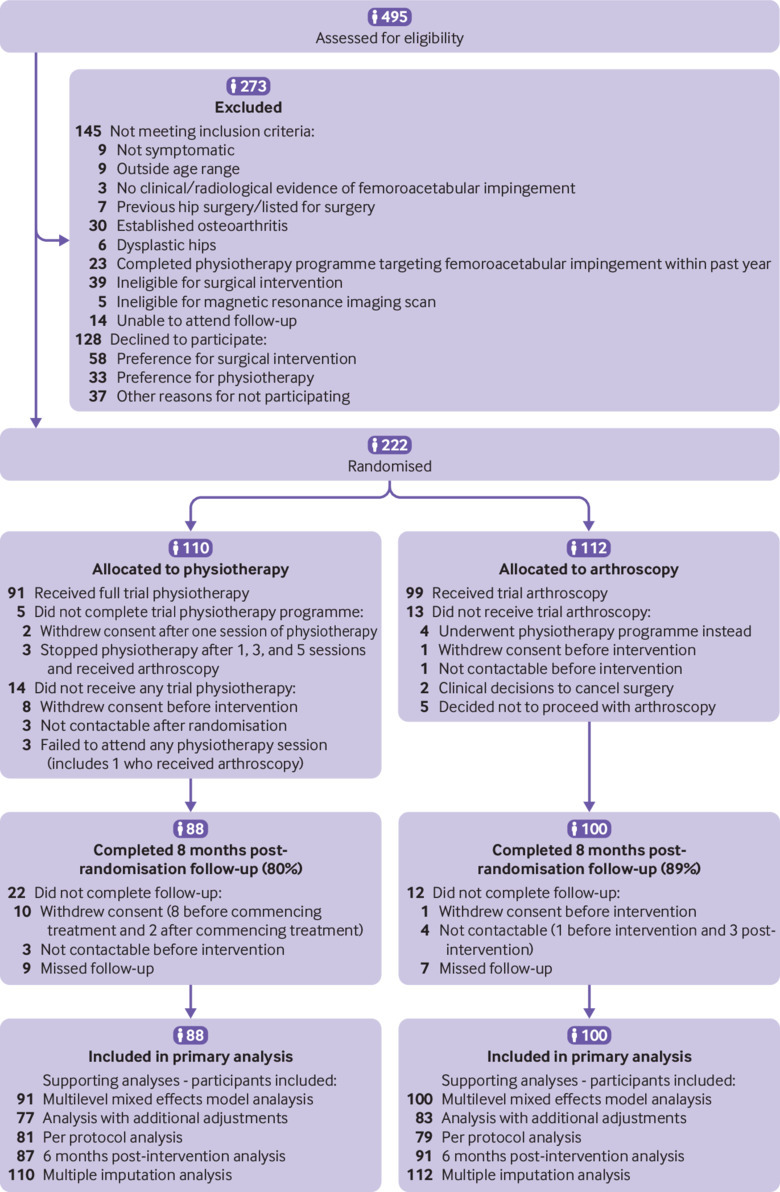
CONSORT diagram

Of 495 patients screened across seven orthopaedic centres between 24 May 2013 and 30 September 2016, 350 (71%) met the study eligibility criteria (fig 3). Of the 350 eligible patients, 222 (63%) elected to participate (45% of all patients screened) and were randomised to arthroscopic surgery (n=112) or to a physiotherapy programme (n=110). The principal reason for declining participation was treatment preference for surgery (n=58, 45%) or for physiotherapy (n=33, 26%). Baseline demographic and clinical characteristics were well balanced across treatment groups (table 1). Mean age was 36.2 years (SD 9.7 years) and there was a higher proportion of women than men (66% *v* 34%). The primary pathology was isolated cam morphology FAI (94%), and the mean baseline HOS ADL was 65.9 (SD 18.7).

**Table 1:** Baseline characteristics of participants. Values are numbers (percentages) unless stated otherwise

Characteristics	Physiotherapy programme* (n=110)	Arthroscopic surgery (n=112)	Total (n=222)
Affected hip:			
Left	51 (46)	45 (40)	96 (43)
Right	59 (54)	67 (60)	126 (57)
Sex:			
Men	37 (34)	38 (34)	75 (34)
Women	73 (66)	74 (66)	147 (66)
Age (years):	n=110	n=112	
Mean (SD)	36.0 (9.9)	36.4 (9.6)	36.2 (9.7)
Range	18-60	18-59	18-60
Height (cm):	n=107	n=111	
Mean (SD)	171.9 (9.2)	170.5 (10.4)	171.2 (9.8)
Range	154-193	151-211	151-211
Weight (kg):	n=108	n=109	
Mean (SD)	78.6 (14.6)	76.1 (18.7)	77.3 (16.8)
Range	53-117	42-143	42-143
Body mass index:	n=106	n=109	
Mean (SD)	26.6 (4.8)	25.9 (4.8)	26.2 (4.8)
Range	18-41	17-42	17-42
Baseline HOS ADL:	n=110	n=112	
Mean (SD)	65.7 (18.9)	66.1 (18.5)	65.9 (18.7)
Range	12-99	28-99	12-99
Morphology:			
Cam	104 (94)	104 (93)	208 (94)
Pincer	0 (0)	1 (0.9)	1 (0.5)
Mixed	6 (5)	7 (6)	13 (6)
**α angle variables**			
Bone average:	n=95	n=94	
Mean (SD)	66.8 (11.8)	67.4 (12.5)	67.1 (12.2)
Range	43-93	43-112	43-`112
Bone maximum:	n=95	n=94	
Mean (SD)	86.4 (16.9)	85.9 (17.1)	86.1 (17.0)
Range	46-128	47-120	46-128
Cartilage average:	n=95	n=94	
Mean (SD)	67.2 (10.8)	67.4 (11.5)	67.3 (11.1)
Range	47-90	46-110	46-110
Cartilage maximum:	n=95	n=94	
Mean (SD)	86.3 (15.5)	85.6 (15.4)	86.0 (15.4)
Range	50-120	49-118	49-120
Lateral centre-edge angle:	n=105	n=106	
Mean (SD)	29.2 (6.7)	28.5 (6.8)	28.8 (6.8)
Range	13-51	15-53	13-53
Kellgren-Lawrence grade†:			
0	87 (79)	90 (80)	177 (80)
1	18 (16)	16 (14)	34 (15)
No radiograph	5 (4)	6 (5)	11 (5)

* Includes activity modification.

† Severity of osteoarthritis.

In the arthroscopic surgery group, 99 (88%) participants received their allocated treatment, and in the physiotherapy programme group, 96 (87%) participants commenced and 91 (83%) completed their allocated treatment (table 2 and fig 3). Of the 19 participants who did not complete their allocated physiotherapy programme, 10 withdrew from the study (eight before intervention and two after the first physiotherapy session), three were not contactable after randomisation, three decided to stop physiotherapy after commencing treatment and subsequently received arthroscopic surgery, and three failed to attend physiotherapy appointments.

**Table 2:** Details of participants commencing allocated intervention. Values are numbers (percentages) of participants unless stated otherwise

Variables	Arthroscopic surgery (n=99)	Physiotherapy programme* (n=96)
Time from randomisation to surgery or starting physiotherapy (days):		
Median (interquartile range)	86 (59-132)	44 (33-61)
Range	5-435	14-251
**Physiotherapy programme†**		
No of sessions attended:		
Median (interquartile range)	–	6 (4-8)
Range	–	1-8
Duration of first session (mins):	–	
Median (interquartile range)	–	60 (60-60)
Range	–	30-95
Duration of follow-up sessions (mins):	–	
Median (interquartile range) (n=83)		30 (30-30)
Range	–	20-60
**Surgical intervention**		
Labral procedure only‡	9 (9)	–
Femoral osteochondroplasty	66 (67)	–
Acetabular osteochondroplasty (rim-trim)	5 (5)	–
Femoral osteochondroplasty+acetabular osteochondroplasty (rim-trim)	19 (19)	
No labral procedure	4 (4)	–
Labral repair	70 (70)	–
Labral debridement	25 (25)	–
No microfracture	90 (90)	–
Microfracture	9 (9)	–
No of physiotherapy sessions attended:		
Median (interquartile range)	4 (2.5-6)	–
Range	1-14	–
Operation time (n=77):		
Median (interquartile range)	55 (45-80)	–
Range	22-160	–

* Includes activity modification. Five patients commenced but did not complete the programme.

† Information available for 88 of 91 patients who completed the physiotherapy programme.

‡ Greater degree of osteoarthritis found at arthroscopy than was evident preoperatively, and no osteochondroplasty performed in three patients. In six patients there was no evidence of femoroacetabular impingement on intraoperative assessment.

Overall, 133 participants (47 arthroscopic surgery and 86 physiotherapy programme) commenced treatment within 12 weeks of randomisation and were assessed at eight months post-randomisation. Intervention started 12 weeks or more after randomisation for 62 participants (52 arthroscopic surgery and 10 physiotherapy programme) and outcomes were measured eight months post-randomisation and six months post-intervention. The substantial proportion of participants who began treatment after 12 weeks reflected increased NHS waiting times within the duration of this study. The median time from randomisation to surgery in the arthroscopic surgery group was 86 days (interquartile range 59-132) and from randomisation to the first appointment in the physiotherapy programme group was 44 (33-61) days (table 2).

Complete data for the primary analysis was available for 188 (85%) participants (88 (80%) of those randomised to the physiotherapy programme and 100 (89%) of those randomised to arthroscopic surgery). Reasons for exclusion of the 34 participants from the primary analysis were loss to follow-up (n=7, 3%), complete withdrawal from trial (n=11, 5%), and incomplete primary endpoint data (n=16, 7%; fig 3).

**Fig 4:**
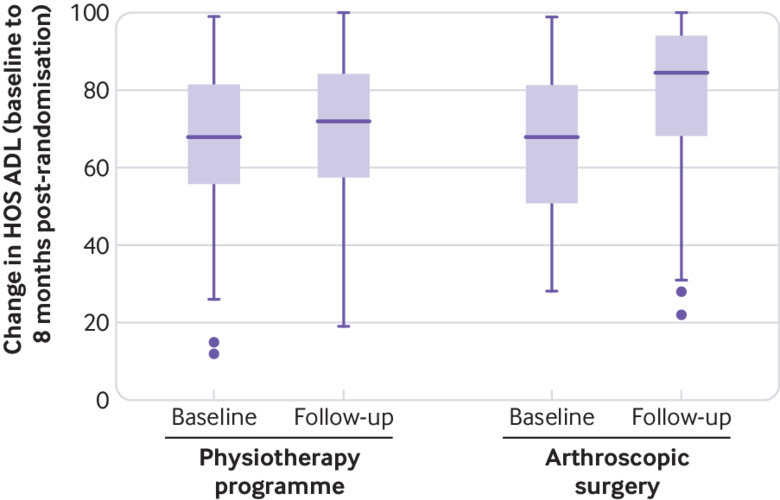
Hip outcome score on activities of daily living subscale (HOS ADL) at baseline and eight months post-randomisation (modified intention to treat). Dots represent extreme outliers

The mean HOS ADL in the arthroscopic surgery group was 10.0 points (95% confidence interval 6.4 to 13.6, P=0.001) higher than in the physiotherapy programme group at eight months post-randomisation. This mean difference was statistically significant and exceeded the prespecified minimum clinically important difference of 9 points, although the lower boundary of the confidence interval was less than 9 points (table 3 and fig 4). Scores on the HOS ADL at eight months post-randomisation were higher than baseline scores in 70% (95% confidence interval 61% to 79%) of participants allocated to arthroscopic surgery compared with 50% (40% to 60%) of those allocated to the physiotherapy programme. Clinically important improvement within the individual, defined as an increase in HOS ADL of at least 9 points, was reported in 51% (41% to 61%) of participants allocated to arthroscopic surgery and 32% (22% to 42%) of those allocated to the physiotherapy programme. A patient acceptable symptomatic state (PASS), defined as HOS ADL greater than 87 points,^27^ was achieved in 48% (38% to 58%) of participants allocated to arthroscopic surgery and 19% (95% confidence interval 11% to 28%) of those allocated to the physiotherapy programme eight months post-randomisation. The proportion of participants who achieved their expectation HOS ADL eight months post-randomisation was 31% (22% to 41%) for arthroscopic surgery and 15% (7% to 22%) for the physiotherapy programme.

**Table 3:** Primary and supporting analyses

Analyses	Physiotherapy programme*			Arthroscopic surgery			Arthroscopic surgery v physiotherapy programme: adjusted† treatment effect (95% CI)	P value
	Mean (SD)	No of patients		Mean (SD)	No of patients			
Primary analysis: HOS ADL 8 months post-randomisation	69.2 (19.1)	88		78.4 (19.9)	100		10.0 (6.4 to 13.6)	<0.001
Analysis A: multilevel mixed effects model‡	-			-			10.5 (6.4 to 14.6)	<0.001
Analysis B: additional adjustment§	69.0 (19.5)	77		80.1 (18.7)	83		11.7 (9.4 to 14.1)	<0.001
Analysis C: per protocol population¶	69.7 (18.6)	81		80.5 (18.9)	79		11.9 (6.2 to 17.5)	0.002
Analysis D: post-intervention analysis**	69.2 (19.3)	87		80.4 (19.6)	91		12.0 (7.3 to 16.7)	<0.001
Multiple imputation analysis	68.0 (20.4)	110		78.2 (20.6)	112		10.0 (5.3 to 14.7)	0.004

* Includes activity modification.

† All analysis models are adjusted for baseline activities of daily living subscale of the hip outcome score (HOS ADL, continuous), sex, age at randomisation (continuous), and site (using cluster robust standard errors).

‡ Multilevel mixed effects model adjusted for HOS ADL, sex and age at randomisation, and time from randomisation (continuous), together with a quadratic term. Participant and study site are used as random effects. Data measured up to 10 months post-randomisation was included in analysis. This analysis concerns 330 observations of 191 participants.

§ Primary analysis repeated with additional covariates: centre-edge angle (continuous), maximum α angle (continuous), Kellgren-Lawrence grade (categorical variable with values 0 and 1), and hospital anxiety and depression scale score (anxiety and depression subscales (continuous)).

¶ Primary analysis repeated for per protocol population (participants who received their allocated intervention at least eight weeks before eight month post-randomisation assessment).

** Primary analysis repeated substituting eight month post-randomisation HOS ADL with six month post-intervention HOS ADL in participants where time from randomisation to intervention exceeded 12 weeks.

Results of all supporting analyses of the HOS ADL, including the per protocol analysis and analysis using multiple imputation were similar to those of the primary analysis, with slightly increased treatment effects that were all statistically significant (table 3). Baseline expectation HOS ADL was not statistically significant when included as a covariable in the primary analysis, and it did not change the treatment effect. The treatment effects were robust even to sensitivity analyses of extreme data missing not at random, which considered outcomes for those with missing data that were up to 9 points worse than expected in the primary analysis (supplementary fig S2).

Subgroup exploration of binary variables identified no evidence of a differential treatment effect for sex or osteoarthritis grade. The small number of individuals with pincer morphology limited the ability to compare outcomes for different FAI type (pincer versus cam versus mixed) (supplementary fig S3). An interaction between treatment and baseline age was suggested, with a decreasing difference in treatment effect between arthroscopic surgery and the physiotherapy programme with increasing age (supplementary table S3 and fig S4). Baseline HOS ADL did not seem to influence the differential treatment effect between groups (supplementary table S3 and fig S5).

Eight month post-randomisation secondary patient reported outcome measure scores including HOS sports subscale, NAHS, OHS, iHOT, HAGOS, UCLA, PainDetect, EQ-5D, and HADS depression score were significantly higher in participants who received arthroscopic surgery compared with those who received the physiotherapy programme (P<0.05) (table 4). The HADS anxiety score did not differ between treatment groups (P=0.18).

**Table 4:** Secondary analysis of patient reported outcome measures

PROMs	No of participants (No of observations)			Arthroscopic surgery *v* physiotherapy programme: adjusted† treatment effect (95% CI)	P value
	Physiotherapy programme*	Arthroscopic surgery			
HOS sports subscale†	91 (166)	99 (163)		11.7 (5.8 to 17.6)	<0.001
OHS‡	87 (160)	92 (153)		5.3 (3.2 to 7.5)	<0.001
NAHS‡	78 (139)	91 (147)		11.2 (6.8 to 15.7)	<0.001
iHOT‡	88 (162)	92 (155)		2.0 (1.3 to 2.8)	<0.001
HAGOS subscales‡:					
Symptoms	88 (161)	92 (155)		13.3 (8.1 to 18.6)	<0.001
Pain	88 (161)	92 (154)		12.7 (8.1 to 17.2)	<0.001
Activities of daily living	88 (162)	92 (154)		11.6 (6.7 to 16.6)	<0.001
Sport	88 (161)	92 (155)		13.1 (7.0 to 19.1)	<0.001
Participation in physical activities	88 (162)	91 (153)		14.6 (7.2 to 22.0)	<0.001
Quality of life	88 (162)	91 (154)		13.2 (7.5 to 19.0)	<0.001
UCLA‡	88 (162)	92 (155)		0.6 (0.1 to 1.0)	0.01
PainDetect score§	62 (101)	61 (93)		−2.1 (−4 to −0.2)	0.03
HADS anxiety§	88 (162)	91 (153)		−0.6 (−1.4 to 0.3)	0.18
HADS depression§	88 (162)	91 (153)		−1.3 (−2.2 to −0.4)	0.004
EQ-5D-3L index‡	88 (161)	91 (153)		0.1 (0.0 to 0.1)	0.003
EQ-5D-3L VAS‡	85 (153)	86 (145)		0.7 (0.3 to 1.2)	0.002

HOS=hip outcome score; OHS=Oxford hip score; NAHS=non-arthritic hip score; iHOT=international hip outcome tool; HAGOS=Copenhagen hip and groin outcome score; UCLA=University of California at Los Angeles; HADS=hospital anxiety and depression score; EQ-5D-3L=European quality of life descriptive system; VAS=visual analogue scale.

* Includes activity modification.

† Multilevel mixed effects model for modified intention-to-treat population adjusted for baseline activities of daily living subscale of HOS, sex and age at randomisation, time from randomisation (continuous), together with quadratic term. Participant and study site are used as random effects. Data measured up to 10 months post-randomisation included in analysis.

‡ Higher values indicate better outcomes.

§ Lower values indicate better outcomes.

Patients allocated to arthroscopic surgery had a greater range of hip flexion than those allocated to physiotherapy eight months post-randomisation, although there was no statistically significant difference for other movements (table 5). At follow-up a smaller proportion of patients allocated to arthroscopic hip surgery reported pain on hip flexion compared with those allocated to the physiotherapy programme. This also applied to hip abduction and adduction, and to the FAbER test but not FAdIR test (table 6).

**Table 5:** Range of movement (ROM) in hip at baseline and eight month post-randomisation assessment

Hip movement	Physiotherapy programme		Arthroscopic surgery		Difference in ROM adjusted for baseline (95% CI)	P value
	Baseline	8 month assessment	Baseline	8 month assessment		
Flexion:	n=107	n=85	n=111	n=96		
Mean (SD)	95.7 (19.1)	99.7 (17.5)	96.9 (15.8)	105.8 (16.3)	4.8 (0.5 to 9.1)	0.03
Range	27-126	25-130	50-130	40-138		
Extension:	n=100	n=83	n=104	n=96		
Mean (SD)	17.9 (7.9)	15.7 (8.0)	18.2 (8.0)	16.8 (7.4)	1.6 (−0.6 to 3.8)	0.16
Range	5-50	0-46	0-40	0-45		
Abduction:	n=107	n=84	n=110	n=96		
Mean (SD)	27.5 (11.9)	29.6 (11.7)	27.1 (12.0)	30.3 (10.6)	1.0 (−2.1 to 4.1)	0.53
Range	5-60	5-70	5-80	8-66		
Adduction:	n=104	n=84	n=108	n=96		
Mean (SD)	21.6 (7.9)	23.2 (8.9)	20.9 (8.2)	23.9 (8.2)	1.1 (−1.2 to 3.5)	0.35
Range	5-44	5-50	5-60	9-45		
Internal rotation:	n=107	n=84	n=110	n=96		
Mean (SD)	24.0 (11.2)	28.9 (11.2)	24.9 (11.2)	30.8 (10.6)	1.4 (−1.6 to 4.4)	0.37
Range	5-55	2-55	2-56	5-69		
External rotation:	n=107	n=84	n=110	n=96		
Mean (SD)	25.0 (11.8)	27.4 (9.7)	26.2 (10.6)	27.0 (8.9)	−1.1 (−3.6 to 1.4)	0.38
Range	5-80	8-70	7-80	10-50		

**Table 6:** Hip assessment at baseline and eight month post-randomisation. Values are numbers (percentages) of participants

Hip assessments	Physiotherapy programme			Arthroscopic surgery		P value*
	Baseline (n=110)	8 month assessment (n=110)		Baseline (n=112)	8 month assessment (n=112)	
Pain on flexion:						
Yes	77 (70)	56 (51)		80 (71)	46 (41)	0.01
No	31 (28)	29 (26)		31 (28)	51 (46)	
Not available	2 (2)	25 (23)		1 (1)	15 (13)	
Pain on extension:						
Yes	44 (40)	24 (22)		41 (37)	18 (16)	0.10
No	61 (55)	59 (54)		67 (60)	79 (71)	
Not available	5 (4.5)	27 (25)		4 (4)	15 (13)	
Pain on abduction:						
Yes	72 (65)	48 (44)		74 (66)	41 (37)	0.05
No	36 (33)	36 (33)		38 (34)	56 (50)	
Not available	2 (2)	26 (24)		0 (0)	15 (13)	
Pain on adduction:						
Yes	51 (46)	39 (35)		61 (54)	30 (27)	0.03
No	55 (50)	45 (41)		50 (45)	67 (60)	
Not available	4 (4)	26 (24)		1 (1)	15 (13)	
Pain on internal rotation:						
Yes	78 (71)	47 (43)		77 (69)	44 (39)	0.16
No	30 (27)	37 (34)		34 (30)	53 (47)	
Not available	2 (2)	26 (24)		1 (1)	15 (13)	
Pain on external rotation:						
Yes	55 (50)	33 (30)		50 (45)	30 (27)	0.24
No	53 (48)	51 (47)		61 (54)	67 (60)	
Not available	2 (2)	26 (24)		1 (1)	15 (13)	
FAdIR test result†:						
Positive	95 (86)	66 (60)		103 (92)	70 (63)	0.38
Negative	11 (10)	18 (16)		9 (8)	26 (23)	
Not available	4 (4)	26 (24)		0 (0)	16 (14)	
FAbER test result‡:						
Positive	89 (81)	52 (47)		91 (81)	42 (38)	0.02
Negative	18 (16)	32 (29)		21 (19)	54 (48)	
Not available	3 (3)	26 (24)		0 (0)	16 (14)	

* χ^2^ test for association between outcomes eight months post-randomisation.

† Pain on flexion, adduction, and internal rotation.

‡ Pain on flexion, abduction, and external rotation.

At the eight month follow-up, two patients crossed over to receive arthroscopic surgery on reporting no improvement in symptoms after the physiotherapy intervention (in addition to four patients who were allocated to the physiotherapy programme but received arthroscopic surgery before completing their physiotherapy programme). A further patient in the physiotherapy group was referred to the chronic pain service. Complications occurred in three (3%) patients in the arthroscopic surgery group. Superficial wound infection was reported for one patient 12 days after surgery that resolved with oral antibiotics. Injury to the lateral cutaneous nerve of the thigh was reported for two patients; it had resolved in one patient by the eight month follow-up. No participant had serious adverse events related to the trial intervention or trial procedure.

## Discussion

This trial found that patients with symptomatic femoroacetabular impingement (FAI) experience a greater improvement in symptoms with arthroscopic hip surgery than with physiotherapy and activity modification eight months post-randomisation. The 10 point mean difference in activities of daily living on the hip outcome score (HOS ADL) between groups is greater than the prespecified minimum clinically important difference of 9 points; however, the lower boundary of the confidence interval is less than this 9 point threshold for clinical importance. In this cohort, the difference in HOS ADL between treatment groups is expected to lie between 6.4 and 13.6 points in favour of arthroscopic surgery.

Overall, 51% of participants randomised to arthroscopic surgery and 32% randomised to a programme of physiotherapy and activity modification reported an improvement in HOS ADL of at least 9 points (minimum detectable change and a clinically important change within an individual). In addition, 48% of participants in the arthroscopic surgery group and 19% in the physiotherapy programme group achieved the patient acceptable symptomatic state (PASS) after treatment.

Blinded clinical assessments revealed a greater improvement in the range of hip flexion and associated discomfort in patients allocated to arthroscopic surgery compared with those allocated to the physiotherapy programme. Additional patient reported outcome measures also indicated superior outcomes in patients randomised to arthroscopic surgery.

### Comparison with other studies

Two randomised controlled trials comparing physiotherapy rehabilitation with arthroscopic surgery for symptomatic FAI were published in 2018 with comparable protocols to this study. One trial did not find a difference between arthroscopic surgery and physiotherapy at any time point up to two year follow-up, although there was a 70% crossover from physiotherapy to arthroscopic surgery.^8^ The other trial concluded that arthroscopic surgery was superior to best conservative care in improving symptoms at 12 month follow-up but that it was not cost effective.^9^ Contrary to our study, the investigators did not find differences between treatment groups for secondary outcome measures of general health related quality of life (EQ-5D and SF-12). Arthroscopic surgery and physiotherapy are safe, and the low complication rates found in this trial are consistent with those of other studies.^7^ ^28^ The age and sex of participants recruited reflected national trends in the provision of arthroscopic hip surgery.^11^

### Strengths and limitations of this study

Consultant orthopaedic surgeons with a specialist interest in hip arthroscopy performed the surgery, which reflects the provision of hip arthroscopy in the NHS and recommendations from the National Institute for Health and Care Excellence. Participating centres consisted of five district general hospitals and two university teaching hospitals. The delivery of care by surgeons performing a high volume of arthroscopic hip procedures ensured skill levels beyond the steep learning curve for this surgery, and the risk of complications is higher for surgeons performing a low volume of procedure.^29^ ^30^ A limitation of our study is that most of the participants were recruited from the coordinating centre; however, the treatment effect was consistent for centres recruiting more than 20 participants (supplementary fig S3).

Physiotherapists of different seniority and trained in the study protocol delivered the physiotherapy programme, with a maximum of eight sessions. Little evidence exists to guide the development of an optimal physiotherapy protocol. It could be that a greater number and frequency of physiotherapy sessions with only senior specialist physiotherapists might improve outcomes. To ensure generalisability and restrict excess treatment costs, we compared arthroscopic hip surgery with a physiotherapy intervention that is deliverable within the constraints of NHS resources. Standard commissioning in the NHS limits physiotherapy provision to approximately six sessions of individual physiotherapy, and we offered a maximum of eight sessions.

Patients in both treatment groups received physiotherapy, either as their primary intervention or as post-surgical rehabilitation. It is important to emphasise the difference in these regimens. The focus of physiotherapy for the treatment of symptomatic FAI (FAI syndrome) (randomised study intervention) was to improve pain and function. The principal elements of our programme started with activity and movement modification, followed by muscle strengthening and segmental stabilisation, and finally optimisation of functional movements with sensory motor training and return to activity according to patient goals. This physiotherapy package was delivered over a median of six sessions. The focus of physiotherapy post-arthroscopic surgery was to maintain range of movement and guide return to activity. Patients were advised to commence active range of movement and isometric exercises the day after surgery, progressing to stretches and static bicycle exercise (no resistance) within a week. Strengthening exercises and low impact activities were introduced after three weeks, usually under physiotherapist guidance, and impact exercise was permitted after six weeks, with sport specific rehabilitation when appropriate. This physiotherapy package was delivered over a median of four sessions.

The clinical significance of an improved range of hip flexion in patients allocated to arthroscopic surgery compared with physiotherapy is not known. A cohort study of patients receiving arthroscopic surgery found that hip flexion was the only movement associated with improved patient reported outcome measures.^31^ A possible explanation is the functional importance of this movement during everyday activities such as sitting or climbing stairs, when pain is often experienced with FAI syndrome. Despite the study limitation of multiple statistical tests being carried out, our results also suggest less pain on hip movements in those allocated to arthroscopic surgery compared with physiotherapy and activity modification.

Overall, 70% of participants randomised to arthroscopic surgery and 50% randomised to physiotherapy and activity modification reported an improvement in HOS ADL of at least 1 point; however, only half the participants randomised to arthroscopic surgery reported an improvement in HOS ADL exceeding 9 points or achieved the PASS. A limitation of reported minimally clinically important differences between groups or changes within an individual is that they are specific to the cohort and to the methodology used by the researchers to calculate values. We prespecified an HOS ADL of 9 points as the minimum clinically important difference between groups.^16^ We also used this value to explore the proportion of participants who achieved a clinically important change in HOS ADL. Since developing the study protocol, the smallest detectable change in HOS ADL within an individual has been calculated as 9 points and the minimum clinically important change in HOS ADL within an individual as 5 points.^32^ This finding supports our use of a 9 point threshold to represent both clinically important differences between groups and change within an individual.

Although arthroscopic hip surgery seems superior to physiotherapy and activity modification, patients must be informed of the potential risks and benefits of surgery, including the risk of no improvement. Up to a half of patients may not achieve a clinically important improvement after surgery; hence accurate patient selection is critical to optimising treatment outcomes. Increasing patient age, higher preoperative patient reported scores, and the presence of osteoarthritis have been identified as having a negative impact on outcome in cohort studies of arthroscopic hip surgery.^33^ ^34^ ^35^ ^36^

Exploration of subgroups suggested that older patients might gain less benefit from arthroscopic surgery compared with physiotherapy; however, variation in HOS ADL was large across different ages. Further exploration in a larger population is required to determine the effect of age on outcomes. Cohort studies also report that arthroscopic hip surgery is less effective with increasing age^33^ ^34^; however, older patients also experience improvements in symptoms.^33^

We excluded patients with established osteoarthritis, defined as presence of osteophytes and possible narrowing of joint space width (Kellgren-Lawrence grade 2) or more severe disease. Patients with possible osteophytes and doubtful narrowing of joint space (Kellgren-Lawrence grade 1) were included. Cohort studies suggest that osteoarthritis is only detrimental to outcomes once loss of joint space width has been established.^35^ In our exploratory evaluation of subgroups we did not detect a difference in treatment effect between participants with Kellgren-Lawrence grade 1 disease and those with no radiographic evidence of osteoarthritis (Kellgren-Lawrence grade 0), although our study was not powered for this calculation.

We were unable to explore whether the presence of cam, pincer, or mixed morphology influences treatment effect owing to the small number of patients with pincer impingement. The relative proportion of participants with each FAI type in this cohort reflects the general population, but the results of this study might not be generalisable to pincer and mixed morphology FAI. Exploratory analysis within the study population did not find an association between outcome and any morphological hip measurement, including the magnitude of cam or pincer morphology and an interaction term.

The exclusion of patients with dysplasia and osteoarthritis is a potential limitation of the study given these patients might also benefit from arthroscopic hip surgery. Our inclusion criteria, however, reflect current evidence based clinical practice.^12^ ^13^ We anticipate that advances in imaging will improve our ability to identify patients who are most likely to benefit from intervention and optimise treatment strategies through enhanced diagnosis of osteoarthritis and dynamic assessment of hip morphology. In this study, during surgery, three patients were found to have more advanced osteoarthritis than expected and six patients did not have impingement within a functional range of movement despite the preoperative diagnosis of cam morphology. Planned osteochondroplasty was therefore not performed. Total hip replacement could have been more appropriate in the patients with osteoarthritis.

Psychological factors are likely to influence outcomes from FAI treatment,^32^ as has been shown for joint arthroplasty.^37^ Patient expectation was not found to influence treatment effect in this study, but further exploration into the effect of baseline depression and anxiety on outcomes may be of value, given that cohort studies have shown that they influence outcome.^32^ The most common reason for declining participation was preference for surgery. Four patients randomised to the physiotherapy programme underwent surgery before collection of the primary outcome measure. Our results might in part reflect a nocebo effect of physiotherapy and placebo effect of surgery. The placebo effect has been shown to be large in surgical trials of arthroscopic shoulder decompression^38^ and arthroscopic meniscectomy.^39^ Our blinded clinical assessments offer reassurance of a differential treatment effect between groups. An ongoing trial comparing osteochondroplasty with arthroscopic lavage for FAI syndrome might offer further insight into the efficacy of surgical treatment.^40^

Median time to treatment post-randomisation was 44 days for the physiotherapy programme group and 86 days for the arthroscopic surgery group. Comparing operative and non-operative management is challenging given surgical care is usually delivered at a single time point, whereas physiotherapy takes place over weeks or months. The longer waiting times for surgery might influence results. However, this was a pragmatic trial and the care delivered accurately reflects current practice in NHS settings. We selected intention-to-treat analysis rather than post-intervention analysis as the primary outcome because although groups are balanced at the time of randomisation (a requirement for inferring a causal relation between intervention and outcome), this might not be true at any other time point. We also performed a post-intervention analysis (analysis D), which revealed a comparable treatment effect to the modified intention-to-treat analysis (table 3). Dropouts occurred in both treatment groups, and although the study remained adequately powered, baseline scores were slightly lower in the physiotherapy programme group (supplementary table S2). Nevertheless, our primary analysis adjusts for prognostic factors, and the treatment effect was robust to different assumptions about missing data (missing at random and missing not at random) in our sensitivity analysis (supplementary fig S2).

This trial does not capture patients with minimally symptomatic FAI, a condition that is typically diagnosed and treated in primary care. Instead it provides guidance for the treatment of patients who are referred to secondary or tertiary care with more severe or prolonged symptoms. Given the potential complications of surgery and observed clinical improvement with the physiotherapy programme, we currently recommend physiotherapy as first line treatment. If symptoms continue then the likelihood of symptom improvement with arthroscopic surgery should be considered.

### Conclusions and policy implications

The results of this study suggest that patients with symptomatic FAI referred to secondary or tertiary care achieve a greater improvement in patient reported outcomes with arthroscopic surgery than with a programme of physiotherapy and activity modification. However, further research is required to identify patients most likely to benefit from intervention. The evaluation of treatment cost effectiveness and disease modifying potential with long term follow-up of this cohort will further guide treatment and commissioning decisions.

**What is already known on this topic**

Femoroacetabular impingement (FAI) can cause hip pain (FAI syndrome) and is thought to be responsible for up to half of all hip osteoarthritis
The treatment of FAI remains controversial—physiotherapy and arthroscopic surgery can both improve symptoms, but it is uncertain which treatment is superior
Despite the absence of evidence to support the use of arthroscopic hip surgery over non-operative measures, the number of arthroscopic hip procedures performed each year has risen rapidly

**What this study adds**

This study suggests that arthroscopic hip surgery is superior to physiotherapy and activity modification at improving symptoms in patients referred to secondary or tertiary care with FAI syndrome
Not all patients benefit from surgery, and the decision to operate must follow a detailed discussion between patients and surgeons
The results inform management decisions made by patients, clinicians, and policymakers, but further research is required to identify patients most likely to benefit from intervention

## Web extra

Extra material supplied by authors

## Notes

Contributors: AJRP and SGJ designed the study, and the protocol was developed with VAG, IR, SJD, SW, TCBP, AWM, KLB, AJMDA, AJC, and DJB. IR and SJD performed the statistical analyses. AJRP, VAG, SF, RM, SW, VK, TCBP, AJMDA, and SGJ recruited patients and acquired data. AJRP, VAG, SF, IR, and SGJ drafted the manuscript. All authors revised manuscript drafts, approved the final manuscript, and contributed intellectually important content. SGJ attests that all listed authors meet authorship criteria and that no others meeting the criteria have been omitted. SGJ is the guarantor of the paper and takes responsibility for the integrity of the work from inception to published article.

Ethical approval: The trial protocol was approved by Health Research Authority, National Research Ethics Services Committee South Central–Berkshire (REC reference: 13/SC/0154) and local research and development departments at each participating site.

Data sharing: Anonymised patient level data can be made available on reasonable request after approval from the trial management committee and after signing a data access agreement. Proposals should be directed to the corresponding author. Consent was not obtained for data sharing, but the presented data are anonymised and the risk of identification is low.

Transparency: The lead author (SGJ) affirms that the manuscript is an honest, accurate, and transparent account of the study being reported; that no important aspects of the study have been omitted; and that any discrepancies from the study as planned have been explained.
