# Arthroscopic hip surgery compared with physiotherapy and activity modification for the treatment of symptomatic femoroacetabular impingement: multicentre randomised controlled trial

**DOI:** 10.1136/bmj.m3715

**Published:** 2021-01-18

In this paper by Palmer and colleagues (*BMJ* 2019;364:l185, doi:10.1136/bmj.l185, published 7 February 2019), the international hip outcome tool iHOT-33 (iHOT) and EQ-5D Visual Analogue Scale (VAS) were presented on a 0-10, instead of a 0-100 scale. As such, the adjusted treatment effects for these secondary outcomes, presented in table 4, should read 20.4 (95% confidence interval 13.2 to 27.6) [not 2.0 (95% confidence interval 1.3 to 2.8)] for iHOT-33, and 7.5 (95% confidence interval 2.7 to 12.3) [not 0.7 (95% confidence interval 0.3 to 1.2)] for EQ-5D-VAS. The corresponding P values remain unchanged.

Updates for supplementary table S2

**Table:** 

	Physiotherapy participants in mITT population	Physiotherapy participants excluded from mITT population	Arthroscopy participants in mITT population	Arthroscopy participants excluded from mITT population	Total participants Participants in mITT population	Total participants Participants excluded from mITT population
iHOT$	38.0 (21.6) [2, 94], n=88	26.5 (18.0) [4, 58], n=22	35.0 (22.1) [1, 81], n=100	36.9 (20.8) [9, 80], n=12	36.4 (21.9) [1, 94], n=188	30.2 (19.4) [4, 80], n=34

$Mean (standard deviation) [range].

