# Supplementary material for: Arthroscopic hip surgery compared with physiotherapy and activity modification for the treatment of symptomatic femoroacetabular impingement: multicentre randomised controlled trial
Source: BMJ. 2019 Feb 7;364:l185. doi: 10.1136/bmj.l185 (PMC6365841; doi:10.1136/bmj.l185)

Supplementary Tables and Figures:

Table S1: Recruitment and Treatment by Centre

	**Centre 1 (N)**	**Centre 2 (N)**	**Centre 3 (N)**	**Centre 4 (N)**	**Centre 5 (N)**	**Centre 6 (N)**	**Centre 7 (N)**	**Total (N)**
**Recruitment by centre**	148	32	6	19	8	8	1	222
**Received Arthroscopic Surgery**	65	16	2	9	2	5	0	99
**Treating Surgeon**	1 (Consultant)	2 (Consultant)	3 (Consultant)	1 (Consultant)	1 (Consultant)	1 (Consultant)	1 (Consultant)	10
**Received Physiotherapy**	62	12	4	5	4	3	1	91
**Treating Physiotherapist**	1 (Band 8)<br>5 (Band 7)<br>8 (Band 6)	1 (Band 7)	1 (Band 7)	1 (Band 7)	2 (Band 6)	1 (Band 7)	1 (Band 7)	21

Table S2: Comparison of baseline data for participants with and without available data for the primary endpoint

	**Physiotherapy**<br><br>Participants in mITT population	**Physiotherapy**<br><br>Participants excluded from mITT population	**Arthroscopy**<br><br>Participants in mITT population	**Arthroscopy**<br><br>Participants excluded from mITT population	**Total Participants**<br><br>Participants in mITT population	**Total Participants**<br><br>Participants excluded from mITT population
**Participants**	88	22	100	12	188	34
**Male***	30 (34%)	7 (32%)	32 (32%)	6 (50%)	62 (33%)	13 (38%)
**Age (years)** $	36.4 (10.3) [18, 60], n=88	34.2 (7.7) [21, 48], n=22	36.2 (9.8) [18, 59], n=100	38.2 (8.6) [24, 49], n=12	36.3 (10.0) [18, 60], n=188	35.6 (8.1) [21, 49], n=34
**BMI**$	26.8 (5.0) [18, 41], n=87	25.5 (3.9) [18, 31], n=19	25.8 (4.7) [17, 42], n=97	26.7 (6.0) [20, 39], n=12	26.3 (4.9) [17, 42], n=184	26.0 (4.8) [18, 39], n=31
**HOS ADL**$	67.5 (18.9) [12, 99], n=88	58.7 (17.5) [28, 93], n=22	66.2 (18.7) [28, 99], n=100	65.5 (18.2) [37, 97], n=12	66.8 (18.8) [12, 99], n=188	61.1 (17.8) [28, 97], n=34
**HOS Sports**$	49.7 (22.7) [0, 94], n=88	39.0 (21.4) [6, 75], n=22	48.5 (22.6) [0, 100], n=99	52.1 (29.3) [0, 100], n=12	49.1 (22.6) [0, 100], n=187	43.6 (24.9) [0, 100], n=34
**OHS**$	29.7 (9.6) [5, 47], n=87	23.1 (11.1) [8, 40], n=22	28.6 (9.5) [8, 45], n=100	27.0 (6.0) [16, 38], n=12	29.1 (9.6) [5, 47], n=187	24.5 (9.7) [8, 40], n=34
**iHOT**$	3.8 (2.2) [0, 9], n=88	2.6 (1.8) [0, 6], n=22	3.5 (2.2) [0, 8], n=100	3.7 (2.1) [1, 8], n=12	3.6 (2.2) [0, 9], n=188	3.0 (1.9) [0, 8], n=34

*Frequency and percentage $Mean (Standard Deviation) [Range]

Table S3: Subgroup exploration of the effect of age and baseline HOS ADL on eight-month post-randomisation HOS ADL.

		Coefficient	95% CI	p-value
Treatment*age	Treatment	21.11	15.81 to 26.41	<0.001
	Age	-0.03	-0.11 to 0.06	0.448
	Interaction term	-0.31	-0.44 to -0.18	0.001
Treatment*baseline HOS ADL	Treatment term	22.41	5.29 to 39.53	0.019
	Baseline HOS ADL	0.76	0.64 to 0.88	<0.001
	Interaction term	-0.19	-0.41 to 0.03	0.084

In the interaction model for age (continuous variable), the individual effect of arthroscopic surgery versus physiotherapy is estimated as 21.1 (95% CI 15.8 to 26.4, p-value < 0.001), the individual effect of age is estimated as -0.03 (95% CI -0.11 to 0.06, p-value = 0.448), and the interaction effect between the two variables (change for each one unit increase in age in the arthroscopy group) is estimated as 0.3 (95% CI -0.4, to -0.2, p-value = 0.001). This indicates that differences in HOS outcomes may be greater and in favour of arthroscopy for younger participants, with this effect decreasing as participants get older. In a scatter plot, the line of best fit suggests that the difference in outcome between treatments may decline with increasing age (Figure S4). In the interaction model for baseline HOS ADL (continuous variable), the individual effect of arthroscopic surgery versus physiotherapy is estimated as 22.4 (95% CI 5.3 to 39.5, p-value = 0.019), the individual effect of baseline HOS ADL is estimated as 0.8 (95% CI 0.6 to 0.9, p-value < 0.001) and the interaction effect between the two variables (change for each one unit increase in baseline HOS ADL in the arthroscopy group) is estimated as -0.2 (95% CI -0.4, to 0.0, p-value = 0.084). This indicates that there is no evidence that differences in HOS outcomes differ based on participant baseline HOS ADL score. In a scatter plot, the line of best fit suggests that the difference in outcome between treatments does not change with increasing baseline HOS ADL (Figure S5). The interaction models were adjusted for all covariates included in the primary analysis model. The addition of non-linear terms did not improve the model fit over linear treatment*baseline variable terms.

Figure S1: Imaging Measurements

The presence of osteoarthritis was assessed on an anteroposterior pelvis radiograph using the Kellgren-Lawrence (KL) grade: KL 0 = no radiographic osteoarthritis (A). KL 1 = Possible osteophytes and doubtful narrowing of joint space (B). Osteophytes classically develop at the rim of the acetabulum and femoral neck (yellow arrows). KL 2 = Definite osteophytes and possible joint space width narrowing. These patients were excluded from the study.

Acetabular morphology was evaluated using the lateral centre edge angle on an anteroposterior pelvis radiograph (C & D). A vertical line was drawn from the centre of the femoral head perpendicular to the inter-teardrop line. A line was then drawn from the centre of the femoral head to the lateral sourcil of the acetabulum. The angle between these lines represents the lateral centre edge angle. Values less than 20 degrees suggest dysplasia and these patients were excluded from the study. Values between 20 degrees and 40 degrees are considered normal range (C). Values greater than 40 degrees suggest pincer morphology (D). The centre edge angle was used as a continuous variable in the data analysis.

Cam morphology was quantified using the maximum alpha angle on radial MRI images around the axis of the femoral neck at the 12 o'clock, 1 o'clock, 2 o'clock, and 3 o'clock positions[42]. The 12 o'clock position was taken to be parallel with the femoral shaft diaphysis and the 3 o'clock position was perpendicular to this axis and anterior for both left and right hips. Alpha angle was calculated by drawing a line from the centre of a best-fit circle surrounding the cartilaginous portion of the femoral head to the midpoint of a line transecting the narrowest portion of the femoral neck. A further line was then drawn from the centre of the best-fit circle to where the contour of the femoral head first exits this circle. Radiographic epidemiological studies suggest alpha angles above 60 degrees are elevated and potentially diagnostic. Values were used as a continuous variable in our data analysis. Normal morphology (E) and cam morphology (F).

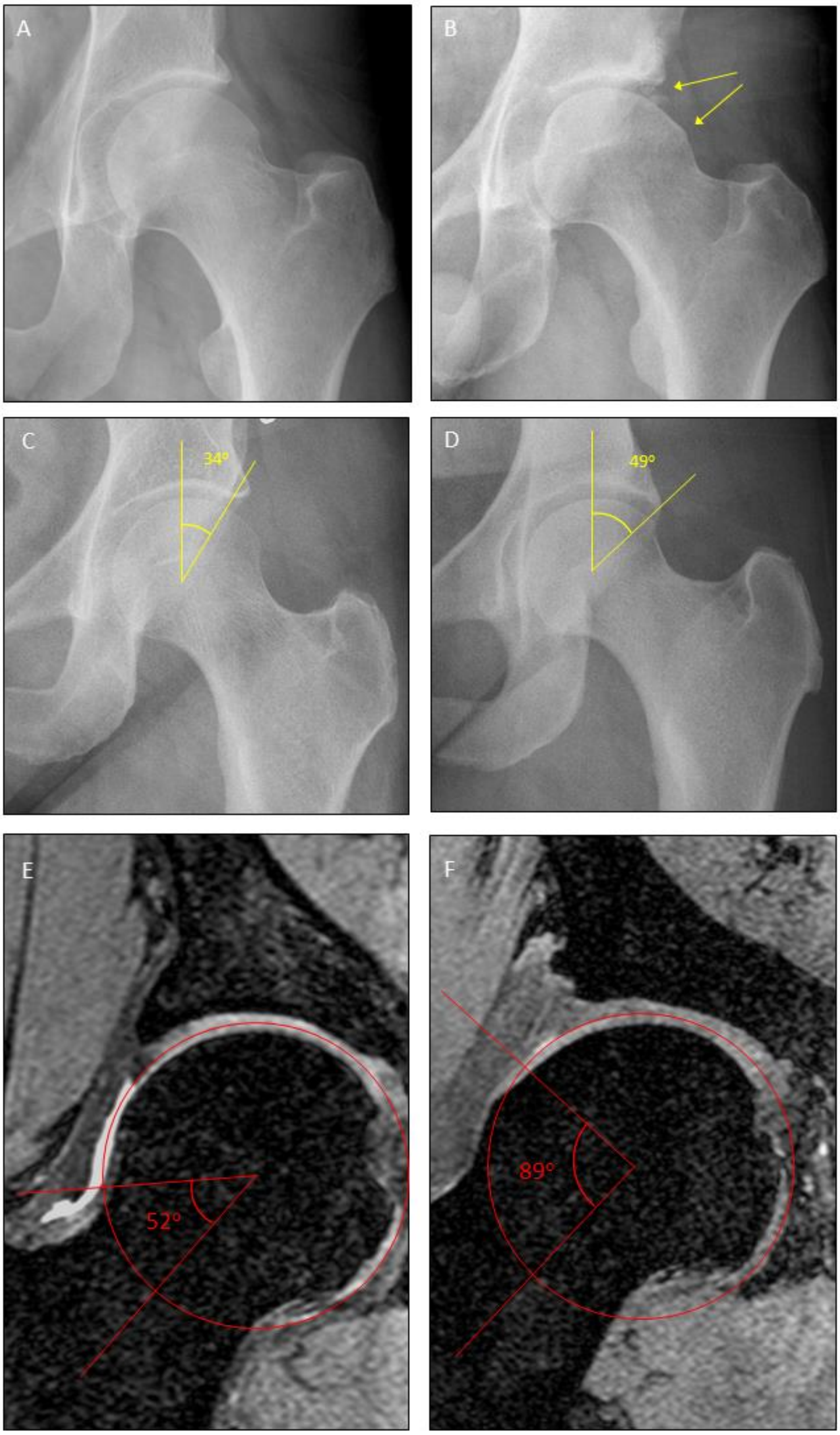
A
B
C
34°
D
49°
E
52°
F
89°

Figure S2: Sensitivity Analysis of the Primary Outcome

Sensitivity analysis for the primary analysis assuming a missing not at random (MNAR) mechanism. Scenarios are considered where participants with missing data in each arm are in turn assumed to differ from missing at random (MAR) by up to 9 points on the HOS ADL (minimally clinically important difference between groups). Departures from MAR are shown on the x-axis. The results from the primary analysis are replicated in the centre of the graph (delta = 0). The results under the estimated MNAR scenarios are shown as treatment effects and 95% CIs. For none of the MNAR scenarios under investigation do the CIs cross zero. Therefore, the trial conclusions do not change for any of the MNAR scenarios considered. The results are therefore robust to plausible departures from the MAR assumption and it is unlikely that the missing data in the outcomes have affected the trial conclusions. More extreme MNAR scenarios, whereby the departure from MAR exceeds 9 were considered very unlikely. However, even for the most extreme MNAR scenarios, the lower limits of the 95% CI are still considerably higher than zero, and therefore the primary analysis is thought to be robust to even more extreme MNAR assumptions.

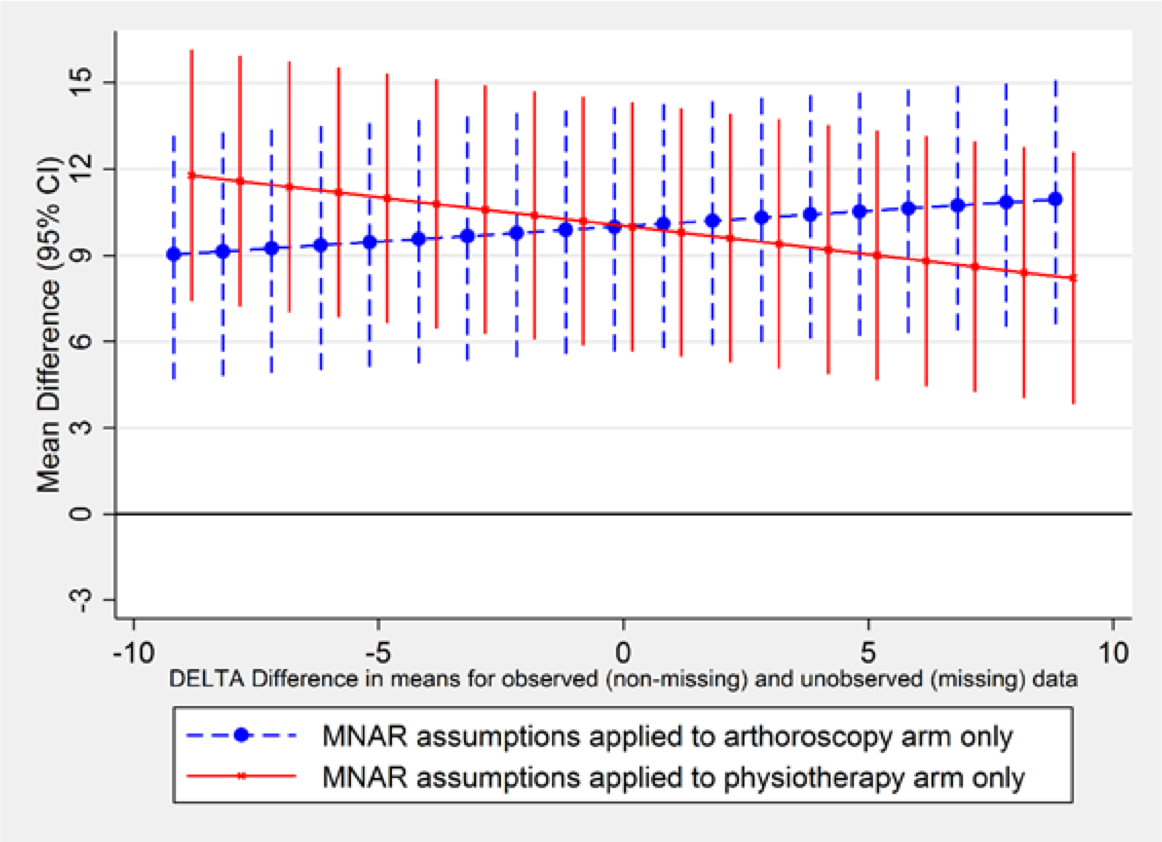

Figure S3: Exploratory Sub-Group Analysis of Gender, Kellgren-Lawrence Grade, Hip Morphology, and Study Centre

HOS ADL at baseline and eight months post randomisation follow up (mITT). Forest plot illustrating treatment effect. Point estimates, 95% confidence intervals, and heterogeneity p values. ES = effect size.

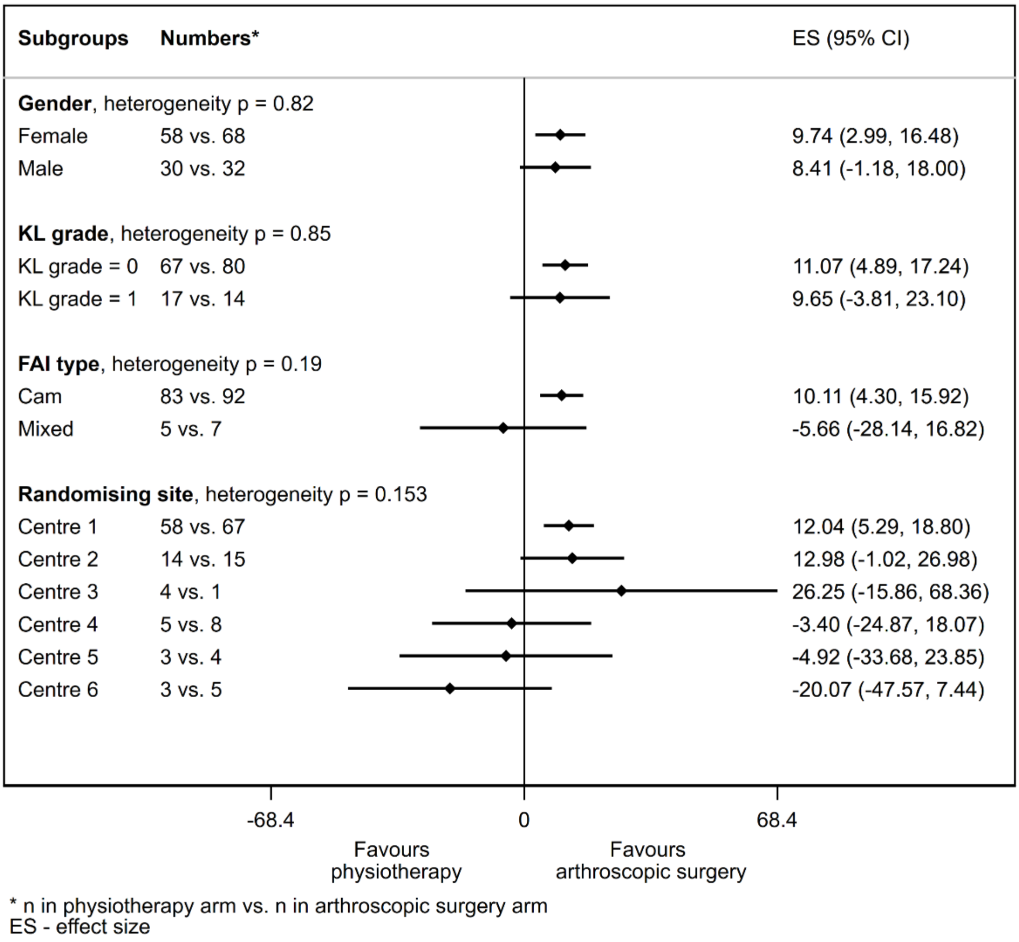

Figure S4: Scatter plot of HOS ADL at eight-months post randomisation versus age at randomisation by randomisation allocation with fitted lines and 95% confidence intervals.

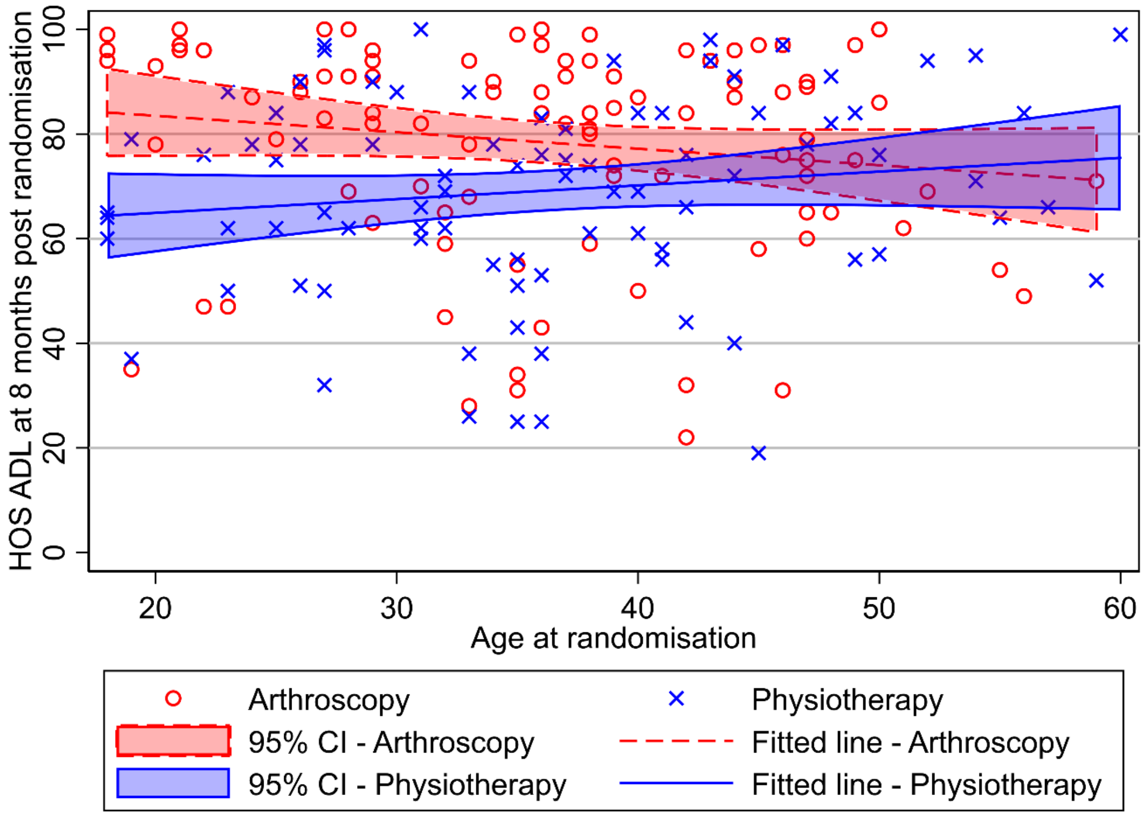

Figure S5: Scatter plot of HOS ADL at eight-months post randomisation versus HOS ADL at randomisation by randomisation allocation with fitted lines and 95% confidence intervals.

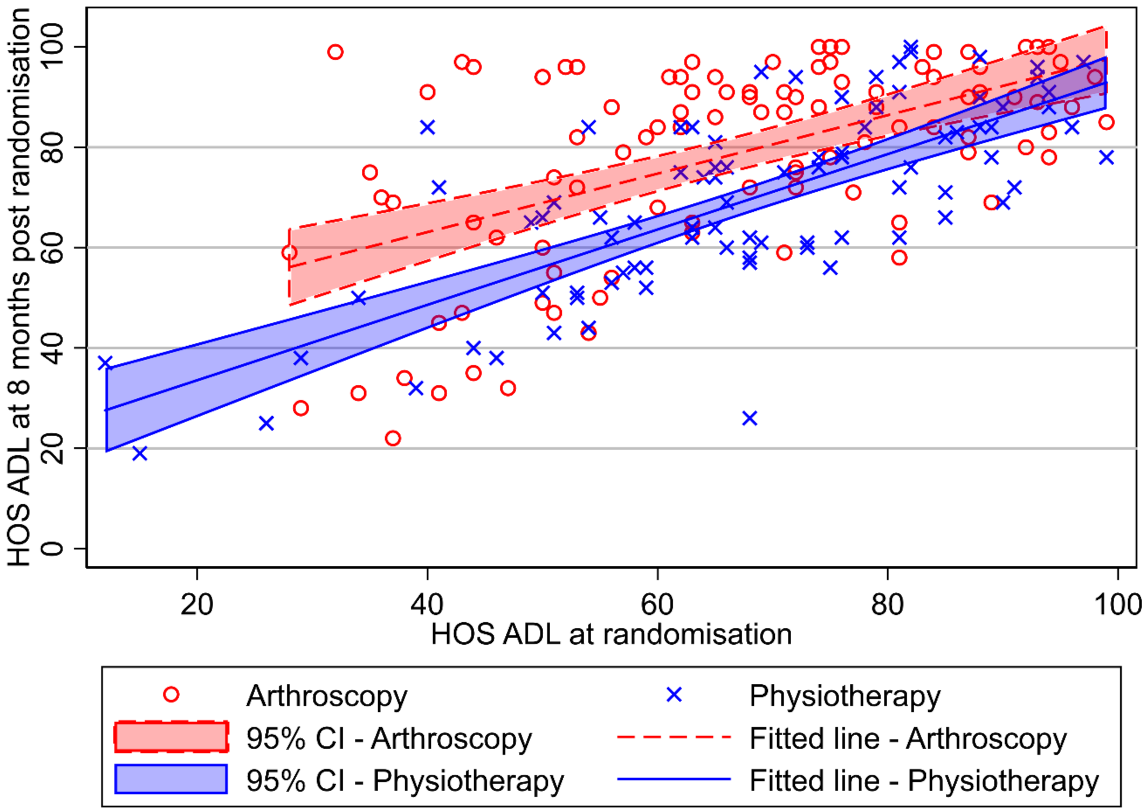

Supplement: Supplementary file 1 — Supplementary information: tables S1-S3 and figures S1-S5 [file pala043530.ww1.pdf]
